# Social adaptability and substance abuse: Predictors of depression among hemodialysis patients?

**DOI:** 10.1186/1471-2369-14-12

**Published:** 2013-01-15

**Authors:** Paulo Roberto Santos, Francisco Plácido Nogueira Arcanjo

**Affiliations:** 1 Sobral School of Medicine, Federal University of Ceará, Avenida Comandante Maurocélio Rocha Ponte, 100 - CEP 62.042-280, Sobral, CE, Brazil

**Keywords:** Depression, Dialysis, End-stage renal disease, Social adjustment, Substance abuse detection

## Abstract

**Background:**

Several aspects linked to social are involved in the onset of depressive feelings. We aimed to find out if social adaptability and substance abuse predict depression among end-stage renal disease (ESRD) patients undergoing hemodialysis (HD).

**Methods:**

We included 145 ESRD patients undergoing HD. Social adaptability was estimated by the Social Adaptability Index (SAI). Substance abuse was defined according to SAI. We screened for depression by applying the 20-item version of the Center for Epidemiologic Studies Depression Scale. A score ≥ 24 classified the patients as depressed. Comparisons between depressed and non-depressed patients were carried out and logistic regression was performed to test gender, age, total SAI, SAI without the substance abuse item, only the substance abuse score and substance abuse as a categorical variable (yes/no) as predictors of depression.

**Results:**

There were 36 (24.8%) depressed patients. There were no differences regarding demographic and laboratory data between the depressed and non-depressed patients. Mean SAI among depressed and non-depressed patients was, respectively, 6.1 ± 1.6 vs. 6.2 ± 1.9 (p=0.901). The percentage of patients with or without substance abuse among depressed patients was, respectively, 13.8% vs. 13.9% (p=1.000). Gender, age, total SAI, SAI without the substance abuse item, only the substance abuse score and substance abuse as a categorical variable did not predict depression.

**Conclusions:**

Social adaptability and substance abuse did not predict depression in HD patients. We propose that aspects related to socioeconomic status not comprised in SAI items should be ruled out as predictors of depression.

## Background

End-stage renal disease (ESRD) patients undergoing hemodialysis (HD) cope with several problems and difficulties related to the disease and its treatment. As a consequence, depressive feelings are common among them [1,2]. We care for HD patients from a low-income region in Brazil. Our patients need all kinds of help to cope with the treatment, like housing for patients living far from the renal unit, provision of food and government financial assistance. So, we are very interested in all aspects of socioeconomic status and their relationship with patient outcomes. Previously, using a validated Brazilian instrument, we were not able to detect differences in prevalence of depression according to economic class [3].

In light of our daily practice of caring for socially disadvantaged patients, the data on the influence of socioeconomic status on depression led to our interest in finding socioeconomic predictors of depression among HD patients [4,5]. Searching for an appropriate instrument, we found the Social Adaptability Index (SAI) [6]. This instrument is a composite index comprising five kinds of social aspects: education level, marital status, employment status, income and substance abuse. As a sum of these five social items, SAI was able to predict death among HD patients and graft loss among kidney recipients [7-9].

Therefore, we aimed to compare the SAI between depressed and non-depressed HD patients and to test the SAI and its substance abuse item separately as predictors of depression.

## Methods

### Sample

The sample was composed of ESRD patients undergoing HD during June 2011 in the only renal unit in the north of Ceará state, northeastern Brazil. The renal unit works in the reference hospital of the region. The criteria for exclusion were age below 18 years and less than three months on dialysis. One hundred forty-five patients were included of a total of 160. Among the patients excluded there were 7 with less than three months on therapy, 5 who refused to participate and 3 under 18 years. All patients were undergoing conventional HD (three sessions of four hours per week) with polysulfone dialyzers (maximum number of reuses=12). The study protocol and informed consent were approved by the ethics committee of Vale do Acaraú University, with which the hospital is associated. All patients gave written consent to participate in the study. Even though not a criterion for exclusion, we determined after the informed consent that none of the patients in the sample had received treatment for depression previously.

### Social adaptability index (SAI)

The index was calculated by the sum of five components: employment status, education level, marital status, substance abuse and income, as described by Goldfarb-Rumyantzev et al. [6]. All components were scored on a scale of 0 to 3, except for income, which was scored 0 to 2. In addition to total SAI, we tested the SAI without the substance abuse item, only the substance abuse score and substance abuse as a categorical variable (yes/no) separately as an independent predictor. The consistency of SAI tested by Cronbach’s alpha was 0.851 for the total scale and within 0.835 and 0.858 for subscales.

### Depression evaluation

We used the 20-item version of Center for Epidemiologic Studies Depression Scale (CES-D) [10]. We have been applying CES-D among HD samples for years and its consistency, tested by Cronbach’s alpha, is within 0.850-0.900. Respondents rate items by recalling the past week and using a three-point response scale, with higher scores indicating the presence and persistence of symptoms. A score ranging from 0 to 60 is calculated by summing the score of each item. We classified depression as a score ≥ 24, as validated by Silveira and Jorge [11].

### Patient data

The demographic data, time on dialysis and underlying etiology of ESRD were assessed in unit records. The underlying kidney disease was classified by clinical criteria and not by histopathology. The laboratory results were those routinely measured in HD patients: creatinine, albumin, hemoglobin, calcium and phosphorus and Kt/V. Kt/V was estimated using a second-generation Daugirdas formula [12].

### Statistical analyses

Comparisons between depressed and non-depressed patients were carried out by the Chi-square test for categorical variables, and by the Student-t and Mann–Whitney tests, when indicated, for continuous variables. Logistic regression was performed to test gender (male as reference category), age, time on dialysis, total SAI, SAI without the substance abuse item, only the substance abuse score and substance abuse (no substance abuse as reference category) as predictors of depression. Statistical significance was considered to be a P value of <0.05. All the statistical analyses were performed using the SPSS version 13.0 program package [13].

## Results

The sample characteristics are shown in Table 1. The results concerning each component of SAI are shown in Table 2. Among 145 patients, there were 36 (24.8%) with depression. The comparisons between depressed and non-depressed patients are shown in Table 3. The mean SAI among depressed and non-depressed patients was, respectively, 6.1 ± 1.6 vs. 6.2 ± 1.9 (p=0.901) (Table 3). The percentage of patients experiencing substance abuse among depressed and non-depressed patients was, respectively, 13.9 vs. 13.8% (p=1.000) (Table 3). Gender, age, time on dialysis, total SAI, SAI without the substance abuse item, only the substance abuse score and substance abuse as a categorical variable were not predictors of depression (Table 4).

**Table 1:** Sample characteristics

**Variables**	
**Gender**	
Male	88 (60.7)
Female	57 (39.3)
**Age**	48.1 ± 15.6
**Etiology of kidney disease**	
Glomerulonephritis	47 (43.1)
Hypertensive nephrosclerosis	32 (29.4)
Diabetic	12 (11.0)
Obstructive	6 (5.5)
Polycistc	5 (4.6)
Lups	1 (0.9)
Undetermined	6 (5.5)
**Time on dialysis** (months)	53.4 ± 51.4
**Social Adaptation Index**	6.21 ± 1.8
**Social Adaptation Index**	5.97 ± 2.1
**without the substance abuse item**	
**Substance abuse**	
Yes	20 (13.8)
No	125 (86.2)
**Depression**	
Yes	36 (24.8)
No	109 (75.2)
**Laboratory**	
Hemoglobin	10.0 ± 1.7
Creatinine	8.7 ± 2.4
Albumin	4.3 ± 0.4
Kt/V	1.8 ± 0.4

**Table 2:** Distribution of sample according to each variable of the Social Adaptability Index

**Variable**	**N**	**%**
**Employment status**		
Unemployed	42	29.0
Retired	97	66.9
Working part time	4	2.7
Working full time	2	1.4
**Education level**		
Did not complete high school	104	71.7
High school graduate	26	18.0
College graduate	12	8.3
Postgraduate study	3	2
**Marital status**		
Not married	39	27.0
Divorced	11	7.8
Married without children	6	4.1
Married with children	89	61.1
**Substance abuse**		
Abusing drugs, alcohol and tobacco	5	3.5
Abusing substances in 2 of 3 categories	4	2.7
Abusing substances in 1 of 3 categories	11	7.6
None	125	86.2
**Income**		
≤$20K/year per household	112	77.2
$20-50K/year per household	25	17.2
≥50K/year per household	8	5.6

**Table 3:** Comparisons between depressed and non-depressed patients

**Variables**	**Depressed**	**Non-depressed**	**P**
**Gender**			
Male	17 (47.2)	71 (65.1)	0.076
Female	19 (52.8)	38 (34.9)	
**Age**	47.0 ±14.6	48.5 ± 16.0	0.627
**Diabetes**			
Yes	7 (19.4)	12 (11.0)	0.253
No	29 (80.6)	97 (89.0)	
**Time on dialysis** (months)	54.3 ± 48.3	55.1 ± 50.5	0.384
**Social Adaptation Index**	6.1 ± 1.6	6.2 ± 1.9	0.901
**Social Adaptation Index without the substance abuse item**	5.8 ± 1.9	6.0 ± 2.2	0.793
**Substance abuse**			
Yes	5 (13.9)	15 (13.8)	1.000
No	31 (86.1)	94 (86.2)	
**Depression score**	30.9 ± 6.4	11.2 ± 6.5	<0.001
**Laboratory**			
Hemoglobin	9.6 ± 1.6	10.1 ± 1.7	0.162
Creatinine	8.2 ± 2.3	8.9 ± 2.4	0.123
Albumin	4.3 ± 0.4	4.4 ± 0.4	0.528
KtV	1.8 ± 0.5	1.8 ± 0.4	0.437

**Table 4:** Logistic regression to detect predictors for depression

**Variable**	**Odds Ratio**	**Confidence Interval**	**P**
**Gender^1^**	2.135	0.944-4.831	0.069
**Age**	1.003	0.975-1.031	0.856
**Time on dialysis**	1.001	0.994-1.008	0.865
**Social Adaptability Index**	0.971	0.765-1.231	0.805
**Social Adaptability Index without the substance abuse item**	0.977	0.822-1.165	0.791
**Only the substance abuse score**	0.963	0.540-1.724	0.898
**Substance abuse^2^**	1.091	0.316-3.764	0.891

^1^Male as reference category.

^2^No substance abuse as reference category.

## Discussion

Our finding of 24.8% depressed patients is in accordance to the overall literature, which reports depression of 20 to 30% among HD patients [1]. We found the same prevalence of depression among men and women, in line with another study and also with our previous research [3,14]. We think the lack of gender difference is due to the powerful stressors associated with HD, which neutralize the gender differences occurring in the general population [15]. Social factors, like male perception of loss of the status of main household breadwinner due to illness, and biological factors, like low level of testosterone, a protective hormone against depression, can also be explanations [16,17].

We aware of divergences about the optimal cut-off of CES-D to classify depression. We chose a cut-off of ≥ 24, which is within the range for the screening of mild depression, described in the literature as being between 16 and 26 [18].

We were surprised that even though the patients come from a low-income area, the mean SAI of our sample is close to that found among American patients [6,7]. This can be due to the fact that socioeconomic disadvantages are risk factors for ESRD worldwide [19].

We were disappointed that SAI was not different between depressed and non-depressed patients, and did not predict depression. Our initial hypothesis of SAI being a predictor of depression was based on data about the relationship of socioeconomic aspects with depression [4,5]. On the other hand, we have to recognize that the links of low SAI score and bad clinical objective outcomes, like death and graft loss, are more easily explained by health care barriers, lower literacy level (negatively influencing treatment adherence), and less social support to help solve problems [9]. Our hypothesis was that all these factors could also be associated with depressive feelings. But when dealing with subjective outcomes such as depression, rather than with objective outcomes like mortality and morbidity, plausibility usually does not ensure existence. The main reason for unexpected results concerning subjective outcomes is the modulation of socioeconomic aspects by personality and ways of coping. Among the mechanisms proposed to explain how socioeconomic status influences clinical outcomes, social support is the best studied concerning depression. There is doubtless an overlap between social adaptability and social support, especially regarding aspects like presence of cohabitants, employment status and education level. On the other hand, some emotional aspects of social support, for instance reliance on friends and family or opportunities for emotional expression, can be independent of social aspects comprised by the SAI. Even when social support is evaluated by a specific tool, like the 24-item Social Provisions Scale, depression among ESRD patients depends more on patients’ personality than on the perceived social support [20]. In this last referenced study, better social support among patients high in the personality trait of “agreeableness” was associated with a decrease in depressive symptoms, whereas social support had little effect on depression change for individuals ranked as low in “agreeableness”.

In clinical practice, there are no widely used and well-validated instruments to assess socioeconomic status able to work as predictor tools in ESRD. So, an instrument like the SAI, validated as a predictor of objective clinical outcomes among HD and transplanted patients, seemed to us a very practical way of using a short instrument also to predict subjective outcomes, such as depression, surely a main outcome among HD patients. Medical monitoring of patients on HD is a hard task and renal units need to work with practical and, if possible, few instruments. Unfortunately, based on our preliminary data, SAI does not predict depression. Granted, our sample is small and from an underdeveloped area, which means there are more young patients and fewer diabetics, with glomerulonephritis being the main cause of ESRD. So, our results may not hold for other more typical samples. In our country, SAI was never tested as a predictor tool for any outcome. We believe our results can stimulate further research using SAI in larger samples with different profiles from ours.

The use of the SAI offered us an opportunity to evaluate an important question in HD patients: substance abuse. In the nephrology area, substance abuse must be highlighted as a risk for ESRD [21,22]. Substance abuse as a prior condition to the beginning of dialysis can be one explanation for the relatively high prevalence of substance abuse among patients undergoing HD. We found 13.8% of the patients admitted to being or having been substance abusers, a number very close to the 19% found by others [23]. Moreover, substance abuse also needs to be studied as an emotion-oriented coping method against stressors and difficulties of ESRD and HD. In our context of a renal unit located in a very poor region, we cannot forget that in the neighborhoods where most of our patients live the use of recreational drugs is very common, as is their sale.

There were limitations of this study, mainly due to the cross-sectional design. Trends of causality related to depression, social adaptability and substance abuse cannot be clarified. Also, no data about duration of substance abuse (previous or after the beginning the dialysis) were collected. A sample from a single renal unit is always a barrier for generalizations, and as previously commented, our sample is quite different from those from developed areas. However, despite these limitations, we emphasize that it is the first time the SAI has been studied in Brazil, which contributes both to stimulate new research in our country using the same instrument and opens the possibility for comparing results across different countries.

## Conclusions

Social adaptability and substance abuse prevalence did not differ among depressed and non-depressed HD patients, and therefore these variables cannot be used as predictors of depression. We propose that aspects related to socioeconomic status not comprised in SAI items should be ruled out as predictors of depression.

## Abbreviations

CES-D: Center for Epidemiologic Studies Depression Scale; ESRD: End-stage renal disease patients undergoing; HD: Hemodialysis; SAI: Social Adaptability Index.

## Competing interests

The authors declare that they have no competing interests.

## Authors’ contributions

PRS was responsible for the conception, design, analysis and interpretation of data. FPNA edited and made critical revision of the manuscript. Both authors read and approved the final manuscript.

## Pre-publication history

The pre-publication history for this paper can be accessed here:

http://www.biomedcentral.com/1471-2369/14/12/prepub

## References

[B1] Cukor D Peterson RA Cohen SD Kimmel PL Depression in end-stage renal disease hemodialysis patients Nat Clin Pract Nephrol 2006 2 678 687 10.1038/ncpneph0359 17124525

[B2] Watnick S Kirwin P Mahnensmith R Concato J The prevalence and treatment of depression among patients starting dialysis Am J Kidney Dis 2003 41 105 110 10.1053/ajkd.2003.50029 12500227

[B3] Santos PR Depression and quality of life of Hemodialysis patients living in a poor region of Brazil Rev Bras Psiquiatr 2011 33 332 337 22189923 10.1590/s1516-44462011000400005

[B4] Gavin AR Walton E Chae DH Alegria M Jackson JS Takeuchi D The associations between socio-economic status and major depressive disorder among Blacks, Latinos, Asians and non-Hispanic Whites: findings from the Collaborative Psychiatric Epidemiology Studies Psychol Med 2010 40 51 61 10.1017/S0033291709006023 19460189 PMC2788678

[B5] Zivin K Bohnert AS Mezuk B Ilgen MA Welsh D Ratliff S Miller EM Valenstein M Kilbourne AM Employment status of patients in the VA health system: implications for mental health services Psychiatr Serv 2011 62 35 38 10.1176/appi.ps.62.1.35 21209297

[B6] Goldfarb-Rumyantzev AS Rout P Sandhu GS Khattak M Tang H Barenbaum A Association between social adaptability index and survival of patients with chronic kidney disease Nephrol Dial Transpant 2010 25 3672 3681 10.1093/ndt/gfq177 20353959

[B7] Sandhu GS Khattak M Rout P Williams ME Gautam S Baird B Goldfarb-Rumyantzev AS Social Adaptability Index: application and outcomes in a dialysis population Nephrol Dial Transplant 2010 26 2667 2674 21257678 10.1093/ndt/gfq789

[B8] Goldfarb-Rumyantzev AS Rout P Sandhu GS Barenbaum A Patibandla BK Narra A Chawla V Williams M Social adaptability index predicts overall mortality in patients with diabetes J Diabetes Complications 2012 26 44 49 10.1016/j.jdiacomp.2011.12.002 22321220

[B9] Garg J Karim M Tang H Sandhu GS DeSilva R Rodrigue JR Pavlakis M Hanto DW Baird BC Goldfarb-Rumyantzev AS Social adaptability index predicts kidney transplant outcome: a single-center retrospective analysis Nephrol Dial Transplant 2012 27 1239 1245 10.1093/ndt/gfr445 22036942

[B10] Radloff LS The CES-D Scale: A Self-Report Depression Scale for Research in the General Population Appl Psychol Measurement 1977 1 385 401 10.1177/014662167700100306

[B11] Silveira DX Jorge MR Propriedades psicométricas da escala de rastreamento populacional para depressão CES-D em populações clínica e não-clínica de adolescentes e adultos jovens Psiq Clin 1998 25 251 261

[B12] Daugirdas JT Second generation logarithmic estimates of single-pool variable volume Kt-V: An analysis of error J Am Soc Nephrol 1993 4 1205 1213 8305648 10.1681/ASN.V451205

[B13] SPSS Inc SPSS for Windows. Version 13 2006 Chicago: SPSS

[B14] Anees M Barki H Masood M Ibrahim M Mumtaz A Depression in hemodialysis patients Pak J Med Sci 2008 24 560 565

[B15] Angst J Gamma A Gastpar M Lépine JP Mendlewicz J Tylee A Gender differences in depression Eur Arch Psychiatr Clin Neurosci 2002 252 201 209 10.1007/s00406-002-0381-6 12451460

[B16] Kimmel PL Psychological factors in adult end stage renal disease patients with hemodialysis: correlates and outcome Am J Kidney Dis 2000 35 132 140 10.1016/S0272-6386(00)70240-X 10766011

[B17] Yousafzai AR Yoysaf M Jan M Badar A Serum testosterone levels in young male patients with major depression J Ayub Med Coll 2000 12 31 32

[B18] Zich JM Attkisson CC Greenfield TK Screening for depression in primary care clinics: the CES-D and the BDI Int J Psychiatry Med 1990 20 259 277 10.2190/LYKR-7VHP-YJEM-MKM2 2265888

[B19] Norris KC Agodoa LY Unraveling the racial disparities associated with kidney disease Kidney Int 2005 68 914 924 10.1111/j.1523-1755.2005.00485.x 16105022

[B20] Hoth KF Christensen AJ Ehlers SL Raichle KA Lawton WJ A longitudinal examination of social support, agreeableness and depressive symptoms in chronic kidney disease J Behavioral Med 2007 30 69 76 10.1007/s10865-006-9083-2 17219057

[B21] Norris KC Thornhill-Joynes M Robinson C Strickland T Alperson BL Witana SC Ward HJ Cocaine use, hypertension, and end-stage renal disease Am J Kidney Dis 2001 38 523 528 10.1053/ajkd.2001.26845 11532684

[B22] Perneger TV Klag MJ Whelton PK Recreational drug use: a neglected risk factor for end-stage renal disease Am J Kidney Dis 2001 38 49 56 10.1053/ajkd.2001.25181 11431181

[B23] Cukor D Coplan J Brown C Friedman S Cromwell-Smith A Peterson RA Kimmel PL Depression and anxiety in urban hemodialysis patients Clin J Am Soc Nephrol 2007 2 484 490 10.2215/CJN.00040107 17699455

